# Effectiveness of an online social support intervention for caregivers of people with dementia: the study protocol of a randomised controlled trial

**DOI:** 10.1186/s13063-017-2097-y

**Published:** 2017-08-29

**Authors:** Alieske E. H. Dam, Marjolein E. de Vugt, Martin P. J. van Boxtel, Frans R. J. Verhey

**Affiliations:** Department of Psychiatry and Neuropsychology, School for Mental Health and Neuroscience/Alzheimer Centre Limburg, Dr. Tanslaan 12 (level 3 | room 3G3.058), P.O. Box 616, 6200 MD Maastricht, The Netherlands

**Keywords:** Online intervention, Social media, Web-based, Dementia, Informal caregiving, RCT

## Abstract

**Background:**

Caregivers of people with dementia (PwD) face burden, feelings of loneliness, and social isolation. Previous studies have shown promising effects of online e-health interventions. Using social media may facilitate support for dementia caregiver networks. In an iterative step-wise approach, a social support tool entitled “Inlife” was developed. This paper describes the design of a study evaluating the effects of Inlife and its process characteristics.

**Methods:**

A mixed-method, randomised controlled trial with 122 caregivers of PwD will be conducted. Participants will be assigned to either the Inlife social support intervention or a waiting-list control group. After 16 weeks, the control group will obtain access to the Inlife environment. Data will be collected at baseline (T_0_) and at 8-week (T_1_), 16-week (T_2_) and 42-week follow up (T_3_). The 16-week follow-up assessment (T_2_) is the primary endpoint to evaluate the results on the primary and secondary outcomes, measured by self-reported questionnaires. The primary outcomes include feelings of caregiver competence and perceived social support. The secondary outcomes include received support, feelings of loneliness, psychological complaints (e.g., anxiety, stress), and quality of life. A process evaluation, including semi-structured interviews, will be conducted to examine the internal and external validity of the intervention.

**Discussion:**

Using a mixed-method design, our study will provide valuable insights into the usability, effectiveness, and factors related to implementation of the Inlife intervention. Our study results will indicate whether Inlife could be a valuable social support resource in future routine dementia care.

**Trial registration:**

Dutch trial register, NTR6131. Registered on 20 October 2016.

**Electronic supplementary material:**

The online version of this article (doi:10.1186/s13063-017-2097-y) contains supplementary material, which is available to authorized users.

## Background

In the upcoming years dementia will become more visible in our society. Not only due to the growing number of people with dementia (PwD), but also because care increasingly occurs at home. The limited available professional workforce and the high economic costs of dementia care place pressure on informal caregivers and on future dementia care providers [1]. The progressive nature of dementia poses stress and physical and emotional health challenges to informal caregivers [2]. There is no available cure for dementia, therefore, caregiver support interventions particularly focus on the maintenance of quality of life and social engagement [3]. Previous studies have shown that psychosocial caregiver interventions have a moderate effect on caregiver health outcomes [4]. Furthermore, caregivers do not always use available services because of the prevailing stigma and experienced threshold associated with asking for support [5, 6]. Therefore, it will become important to develop cost-effective, accessible and innovative caregiver support methods. The Internet might provide a new arena for support because worldwide Internet usage and computer literacy are rapidly growing [7].

Internet interventions require less financial and human resources, and they are easily accessible in the home setting, regardless of distance, time and physical constraints. Previous studies have shown the promising effects of online training and self-management programmes [8, 9]; in particular, e-health interventions that fit caregiver needs and focus on the enhancement of positive experience are more likely to be accepted and strengthen caregiver functioning [10]. Currently, there are challenges associated with research on technology interventions in dementia care, particularly in the development, methodology, effectiveness and implementation of such research [11]. Hence, further studies are needed to investigate the potential of social media tools in providing support for dementia caregiver networks. Therefore, an innovative social support tool entitled “Inlife” was developed and launched in the Netherlands. The Inlife platform is user-friendly and unique because it was developed specifically for caregivers and PwD to lower the observed threshold for asking for support [6] and to increase positive interactions in personal care circles. The present paper describes the design of a randomised controlled trial (RCT) used to evaluate the effectiveness and feasibility of Inlife for caregivers of PwD. The primary study objectives are as follows:

1. To investigate whether the use of Inlife is more effective than treatment as usual for caregiver social support and caregiver competence
2. To evaluate the intervention quality and sampling quality to determine internal and external validity
3. To explore the barriers and facilitating factors associated with use of the Inlife platform and its successful implementation in (clinical) practice

## Methods/design

**Fig. 1:**
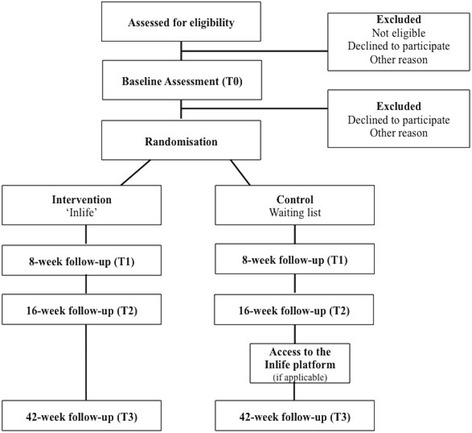
Participant flow diagram according to Consolidated Standards of Reporting Trials (CONSORT)

An RCT design will be used to compare a waiting-list control group with an intervention group. Self-reported data will be collected pre-intervention (T_0_) and at 8-week (T_1_) and 16-week follow up (T_2_). The 16-week follow-up assessment serves as a primary endpoint to compare group effects. After the initial 16-week study period, the participants in the waiting-list control group can start using the Inlife platform, whereas caregivers in the intervention group are free to continue using the platform. Furthermore, a long-term follow-up measurement is scheduled for 42 weeks (T_3_) after baseline. See Fig. 1 for the flow diagram. This paper follows the Standard Protocol Items: Recommendations for Interventional Trials (SPIRIT) guidelines, which can be found in Additional file 1.

### Population

Primary family caregivers of PwD living in the community or in nursing homes will be included in this study. No limits will be set for the type of dementia, type of caregiver relationship or age.

#### Recruitment

Study participants will be recruited from local and national community services (caregiver support services, Alzheimer cafés), Alzheimer societies, written and online advertisements (e.g., Facebook) and through direct registration on the Inlife homepage. Potential study participants will be called, and if they are interested, they will be sent an information letter. They will be contacted again approximately 2 weeks later. When the caregivers are willing to participate, they will be screened in a short telephone interview to confirm the inclusion and exclusion criteria.

#### Eligibility

The following inclusion criteria will be applied: primary caregivers (age 18 years or older) of a PwD (all subtypes), with Internet access, basic (tablet) computer skills and knowledge (as assessed by the researcher), and with at least two social network members who are willing to join the Inlife platform. The exclusion criteria are being overburdened, having serious health problems that could interfere with participation (e.g., burn-out or surgery), as assessed by the study staff, and being unavailable for a full period of more than 4 weeks during the study period.

### Randomisation

Following completion of the baseline assessment, participants will be randomly assigned to either the intervention group, which will use the Inlife platform for 16 weeks or to the waiting-list control group, which will receive treatment as usual. The randomisation will be performed by a computer programme that will be controlled by an independent researcher. A block randomisation procedure will be used to minimise the risk of unbalanced assignment to the intervention and control groups. Randomly generated blocks of several sizes (4, 6 and 8) will be used. The block size and randomisation pattern will be randomly chosen at the beginning of each block. This process reduces the chance that the randomisation process can be predicted. The researchers will remain independent because the allocation and assessments are conducted online. Independent of the group allocation, automatically generated emails (with links for the follow-up assessments) will be sent to the primary caregiver.

### Intervention

#### Intervention condition

Inlife is an online social support platform that consists of several functionalities. The purpose of the platform is to arrange care activities and share daily activities with so-called care-circles to strengthen their social support, positive interactions and feelings of competence in the primary caregiver. The functionalities of Inlife are shown in Table 1.

**Table 1:** Available functionalities in the Inlife app and website

Functionality	Content
Circles	The primary caregiver invites family members, friends and others in three “circles” (i.e., inner, middle and outer), providing the opportunity to attribute distinct levels of privacy and privileges
Profile	Personal information page of the circle members
Timeline	To share positive interactions in daily life with circle members
Personal messages	Personal messages can be shared with individual or several circle members
Helping	An overview of the needs, wishes and offers of support within the care circles
Calendar	A shared schedule to plan daily activities and appointments or to post requests for help
Care book	An overview of the contact details and relevant personal care information
Compass	To navigate to relevant resources regarding dementia and caregiver information

The development process and final intervention are described in more detail elsewhere [12]. A researcher will call the primary caregiver 2 weeks after registering on the Inlife platform, to confirm that the online programme is fully understood. The participants are free to invite as many family members, friends or significant others as they wish into their care circles, and there is no set limit for the frequency of using and posting messages on the Inlife platform.

#### Control condition

Participants in the control group will be placed on a waiting list for 16 weeks. After they complete the 16-week follow-up assessment, they will receive an invitation email to start using the Inlife platform. They will receive the same follow-up measurement that the intervention group received (except for the Programme Participation Questionnaire (PPQ)).

### Procedure

The primary caregivers in the intervention and control groups will be assessed at four time points: the baseline assessment (T_0_), after 8 weeks (T_1_), after 16 weeks (T_2_), and after 42 weeks (T_3_) (i.e., 6 months after T_2_). The waiting-list control group will be invited to start using Inlife after 16 weeks (i.e., following the completion of the T_2_ assessment). At the time of the 42-week follow-up assessment (T_3_), both groups will be exposed to Inlife. Table 2 shows the enrolment and assessment procedures. All data are collected using self-reported measurements that are completed online through a custom, secure and confidential questionnaire system. All primary caregivers will receive a personal link through which they can complete the questionnaires. The participants will have approximately 2 weeks to complete each measurement. If a measurement is incomplete after the first invitation, the participants will receive an automatic reminder email. If the measurements are still incomplete, the researcher will call the participant to request completion of the measurement. Only the involved research staff has access to the trial data. The trial results will be disseminated in publications.

**Table 2:**
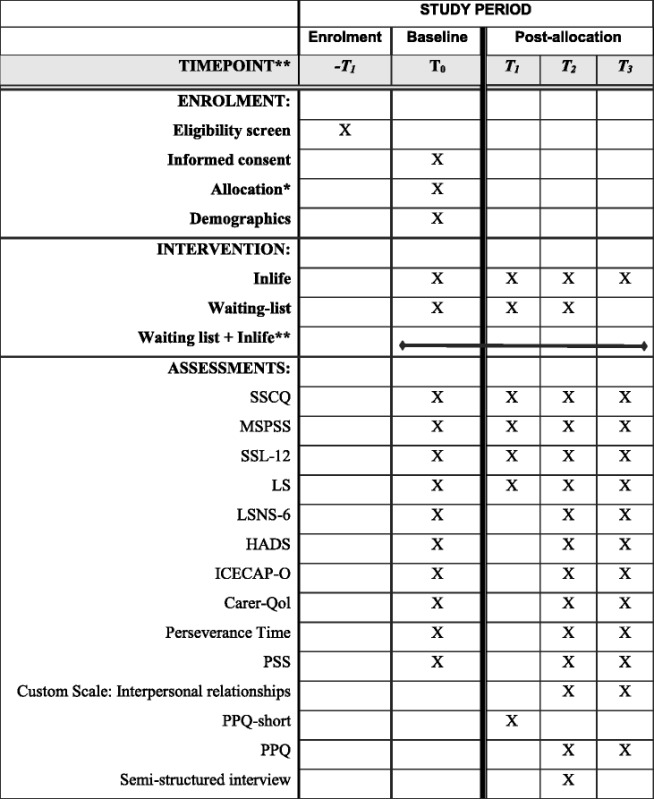
Schedule of enrolment and assessments per condition according to Standard Protocol Items: Recommendations for Interventional Trials (SPIRIT)

*Random allocation takes place after completion of the baseline. **The waiting-list control group starts with Inlife after T_2_. The Inlife intervention group can continue to use the Inlife platform throughout the entire study period. *SSCQ* Short Sense of Competence Questionnaire, *MSPSS* Multidimensional Scale of Perceived Support, *SSL-12* Social Support List 12-Interactions, *LS* Loneliness Scale, *LSNS-6* Lubben Social Network Scale, *HADS* Hospital Anxiety and Depression Scale, *ICECAP-O* Investigating Choice Experiments for the Preferences of Older People Capability measure for Older People, *CarerQol* Care and Quality of Life scale, *PSS* Perceived Stress Scale, *PPQ* Programme Participation Questionnaire

### Participant retention

The study participants will be contacted by phone 2 weeks after their registration on the Inlife platform to reflect on their experiences. Furthermore, the participants will receive a newsletter twice a year to stay informed about the current and future status of the study.

### Measurements

An overview of the different measurements used during the baseline, 8-week, 16-week and 42-week follow-up assessments is shown in Table 2.

#### Demographics

Demographic variables, including the age, sex and educational level of both the caregiver and PwD, will be measured. Additionally, the type and severity of dementia, time since onset of the first complaints, time since the dementia diagnosis, hours of contact with the caregiver and hours of care with/for the PwD will be assessed. A physician, as reported by the caregiver, must have diagnosed dementia. No additional medical information will be requested.

#### Primary outcome measures

The Short Sense of Competence Questionnaire (SSCQ) [13] measures a caregiver's subjective feelings of competence; it consists of 7 items rated on a 5-point Likert scale ranging from 1 (agree very strongly) to 5 (disagree very strongly). The items reflect the level of competence or feelings of being capable of caring for the PwD on three domains: satisfaction with the PwD as a care recipient (3 items), satisfaction with their performance as a caregiver (2 items), and the consequences of involvement in care for the personal life of the caregiver (2 items). A total sum score will be calculated by summing the scores for responses of “disagree” and “strongly disagree” (range 0–7), and higher scores indicate a greater sense of competence [14]. The SSCQ has been widely used in caregiver intervention research, and it has been demonstrated to be valid and reliable [14–16].

The Multidimensional Scale of Perceived Support (MSPSS) [17] is a 12-item scale measuring perceived social support from specific sources on three different subscales of 4 items each: family, friends and significant others. The answers are provided on a 7-point Likert scale ranging from 1 (very strongly disagree) to 7 (very strongly agree). The total score ranges from 12 to 84, and a higher score reflects higher levels of perceived social support. The psychometric properties of the scale are good, as demonstrated in non-clinical [18] and clinical populations [19], and the MSPSS has previously been used in caregiver intervention studies [20].

#### Secondary outcome measures

The Social Support List 12-Interactions (SSL12*-*I) [21] assesses the extent of received social support, as measured in terms of social interaction with members of the primary social network. The questionnaire consists of 12 items divided into 3 subscales of 4 items each: everyday support, social support in problem situations and esteem support. The response options range from 1 (“seldom or never”) to 4 (“often”). The total score ranges from 12 to 48, with higher scores reflecting a higher level of received support. Good internal reliability has been demonstrated [21], and the scale has previously been used in interventions studies for caregivers of PwD [22].

The Loneliness Scale (LS) [23] measures loneliness through perceived isolation or lack of communication with other people. The LS consists of 11 items with 2 subscales: a social subscale (5 items positively worded) and an emotional subscale (6 items, negatively worded). The psychometric qualities are evaluated as sufficient [24]. There are 5 response options, ranging from 1 (“yes! very strongly agree”) to 5 (“no! very strongly disagree”). The total score ranges from 11 to 33, with higher scores indicating greater loneliness.

The Lubben Social Network Scale (LSNS*-*6) [25] is a short instrument to quantitatively assess family and friendship ties and screen for the risk of social isolation using two 3-item subscales (family and friends) asking for either the number of relatives or friends that you see/hear, feel close to or are at ease with, rated on a 6-point scale: 0 (none), 1 (one), 2 (two), 3 (three or four), 4 (five through eight), or 5 (nine or more). The total scale score is an equally weighted sum of the 6 items, ranging from 0 to 30. The scale has been validated in an older sample [25], and it is used in caregiver studies [26].

The Hospital Anxiety and Depression Scale (HADS) [27]. The HADS measures the severity of depression and anxiety symptoms. The anxiety and depression subscales contain 7 items rated on a 4-point Likert scale, ranging from 0 (not at all) to 3 (a great deal of time). The total scores for both scales range from 0 to 21, with higher scores indicating more anxiety and depression symptoms. The HADS has been widely used for caregivers of PwD, and the Dutch version of the HADS has shown good reliability in both normal and clinical populations [28].

The Investigating Choice Experiments for the Preferences of Older People Capability Measure for Older People (ICECAP*-*O) has been used to measure quality-of-life domains [29]. The ICECAP-O assesses a person’s sense of capability and overall wellbeing; measured by five states or attributes associated with attachment, role, enjoyment, security and control. A summary score, ranging from 0 (no capability) to 1 (full capability), is calculated for each statement describing an attribute. Higher scores indicate better wellbeing/quality of life. The ICECAP-O assesses broader outcomes, and compared to the more widely used EQ-5D, it might be more sensitive to differences between the intervention and comparison groups [30].

The Care Related Quality of Life scale (CarerQol) [31] measures the impact of informal care by assessing seven domains of burden (based on the Euroqol-5D (EQ-5D)), which are rated on a 3-point scale ranging from 1 (no burden) to 3 (a lot of burden); it contains an additional valuation component to measure general wellbeing using the degree of happiness scored on a visual analogue scale (VAS), ranging from 0 (completely unhappy) to 10 (completely happy). The CarerQol has been proven to be valid for economic evaluations [32].

Perseverance time is a single item that estimates how long an informal caregiver can maintain current care if the situation remains unchanged, and it is rated using the response to one of four options: (1) less than 6 months, (2) more than 6 months, but less than a year, (3) more than a year, but less than 2 years, or (4) more than 2 years. This questionnaire is validated for informal caregivers of PwD [33].

The Perceived Stress Scale (PSS) will be used to assess the degree to which daily situations are appraised as being stressful. The PSS consists of 10 items rated on a 5-point Likert scale, ranging from 0 (never) to 4 (very often). The sum score on the PSS ranges from 0 to 40, with higher scores representing higher levels of stress. Sufficient psychometric quality has been demonstrated [34].

The caregiver interpersonal relationship scale is a custom questionnaire that aims to measure the degree of positive interactions and connectedness within the caregiver network of the PwD. The authors based the scale on the existing Caregiver Reaction Assessment (CRA) [35] to closely reflect the primary study objective, to increase positive involvement and interactions in the PwD caregiver network. In the literature, there is no validated questionnaire that measures interpersonal relationships through positive interactions and involvement specifically for caregivers of PwD. Although this custom questionnaire has not yet been validated, it provides an approximate indication of the positive interactions experienced by caregivers of a PwD. The scale consists of 6 items rated on a 5-point Likert scale, ranging from 1 (strongly disagree) to 5 (strongly agree). The total score ranges from 6 to 30, with higher scores reflecting more positive interactions within the PwD care network.

The PPQ was adapted for this study to measure the feasibility of Inlife [12]. The PPQ contains 34 items relating to usability, user-friendliness and satisfaction with the Inlife platform; it is measured on a 5-point Likert scale, ranging from 1 (strongly disagree) to 5 (strongly agree). Higher scores reflect greater feasibility. Participants in the intervention group will complete the entire scale after the 16-week intervention period. To minimise burden, a shortened 5-item version of the PPQ will be completed after the 8-week follow up.

### Semi-structured interview

After completion of the 16-week follow-up assessment (T_2_), semi-structured interviews will be conducted by the researchers, with a sub-sample of approximately *n* = 10 primary caregivers in the intervention group. (i.e., depending on the occurrence of saturation in the data) to qualitatively evaluate the usability and user friendliness of the Inlife platform and the effectiveness in social support and feelings of competence. Additionally, these interviews may provide insight into the barriers and facilitating factors for the implementation of Inlife on a larger scale in a real-life setting. The interviews will occur in person at the caregiver’s home or at Maastricht University, and they will be audiotaped (with the participants’ consent). The topics will include the use of the intervention in daily life, potential factors influencing Inlife use, social support impact and feelings of competence in the caregivers.

### Process outcomes

Next to the RCT, a process evaluation will be conducted to improve the interpretation of the results and to explore the factors that might influence the implementation of the Inlife intervention. As part of the process evaluation, the intervention quality and sampling quality will be examined to determine internal and external validity.

Sampling quality will be examined by describing (1) the recruitment procedure, (2) the randomisation and allocation procedure, (3) the informed consent procedure and (4) the barriers and facilitators encountered during the recruitment procedure. The reach will be defined by calculating the proportion of participating caregivers compared to the total number of persons approached.

Intervention quality will be evaluated by determining (1) the relevance of the intervention, (2) the feasibility of the intervention and (3) the extent to which the intervention was performed according to the protocol (e.g., deviations from the Inlife usage period and missing online measurements).

Sampling quality data will be obtained from the research database. Intervention quality data will be collected from the quantitative Inlife Programme Questionnaire, which will be completed after the 16-week intervention period. Subjective experiences will be assessed during the evaluation phone call 2 weeks after the start of the intervention, and from the semi-structured interviews (in a subsample of Inlife users). Additionally, objective measurements of the Inlife user log data (e.g., clicks per functionality and number of new posts), and the number of dropouts will be examined.

### Sample size

The sample size was determined based on previous intervention studies of caregivers of PwD with the Short Sense of Competence Questionnaire (SSCQ) as an outcome measure [16, 36], using repeated measures and interaction within and between variables with a mean effect size of 0.5. In accordance with the following assumptions: alpha 0.05 and power 80%, we aim to include 102 primary caregivers (51 participants per group). Allowing for a 20% loss to follow up, we aim to enrol 122 caregivers.

### Statistical analysis

#### Primary and secondary outcome measures

All analyses will be performed according to the intention-to-treat principle, using IBM SPSS Statistics (IBM Corp. Released 2016. IBM SPSS Statistics for Macintosh, Version 24.0. Armonk, NY: IBM Corp).

Prior to the main analysis, data will be examined for missing values, outliers and normality. To investigate whether randomisation was successful, potential differences in the baseline characteristics of the intervention and control groups will be determined using either the *t* test for the continuous variables or the chi squared (X^2^) test for the categorical variables. In the case of missing values, we will examine whether they are missing completely at random (MCAR). If the variables are MCAR, based on a comparison between the baseline characteristics of the study completers and the participants with missing values (no *P* value < .05), list-wise deletion will be applied, whereas when the missing values are not at random, we will apply a multiple imputation strategy [37].

To test the first hypothesis examining the differences in the outcome variables of the intervention group and waiting-list control group, analysis of covariance (ANCOVA) will be performed, with the outcomes of the T_2_ assessment (i.e., measured at 16 weeks, after this period the waiting-list control group can start with Inlife). The outcomes assessed at the T_2_ follow up will be included in the model as dependent variables and groups as between-subject variables. Potential baseline differences between the treatment arms will be included as covariates, if necessary. Each outcome measured will be assessed as a dependent variable in separate analyses.

To test the changes in the primary and secondary outcome measures over the total study period, data from the intervention group and waiting-list control group will be analysed using a linear mixed model (LMM). The LMM will estimate the fixed effects of the regression slopes representing the changes during the intervals (T_0_-T_1_) and (T_1_-T_2_) in the intervention only and waiting-list control group and after the (T_2_-T_3_) follow up. This process enables a comparison of the rate of change in the caregivers who did not receive the intervention compared to the caregivers who received the intervention (T_0_-T_2_), and it provides insight into whether the potential effects on the outcome variables will be sustained by evaluating the rate of change from the 16-week follow up to the 42-week follow up (T_2_-T_3,_ intervention group only). Furthermore, LMM accounts for the fact that the data are correlated through nesting in individuals. Potential missing values are handled efficiently under the missing at random (MAR) assumption, using maximum likelihood estimates for the missing observations; hence, it is suitable for intention-to-treat analysis. Therefore, variables known to predict missing values will be included in the LMM analyses [38]. Intervals will be entered as categorical variables, using three dummies. The best model fit of the models with random intercept (at the participant level) and with random intercept and random slope (at interval level) will be compared with likelihood ratio tests. All tests will be two-tailed, with an alpha level of 0.05.

### Semi-structured interviews and process evaluation

Data obtained from the qualitative semi-structured interviews (with a subsample of approximately 10 participants) will be transcribed verbatim. Two independent researchers will conduct inductive and deductive content analyses of the transcribed interviews in ATLAS.ti. Text fragments will be assigned codes, which will be organised into categories. Then, the categories will be merged into common themes in a consensus meeting with a third independent researcher.

All participants will rate their opinions regarding the usability and user-friendliness of Inlife on the PPQ. These scores will be analysed through descriptive statistics. Additionally, explorative analyses of the research database will be performed to summarise the response rates, reasons for participation, researchers’ notes of user experiences with Inlife and findings regarding sampling quality and intervention quality.

## Discussion

In this paper, we outlined the design of a randomised waiting-list controlled trial to evaluate the effectiveness and process of Inlife, an online social support platform for caregivers of PwD to improve social support, feelings of caregiver competence and positive interaction. The development of Inlife was based on the Medical Research Council (MRC) framework and tested in a feasibility study [12]. The Inlife intervention is specifically developed with potential users, and it is unique because it facilitates social support for the primary caregiver and PwD in separate care circles, thereby providing different functionalities and privileges to all involved network members.

As suggested by the MRC framework, we considered the factors that can influence implementation by integrating a process evaluation in our study design. The process evaluation will facilitate understanding about the delivery and quality of the intervention and provides insight into the contextual, personal and situational factors influencing the intervention effectiveness in a real-world setting [11, 39]. We expect the intervention to be successfully implemented because Inlife is designed together with potential users, and it will be made available by both community services and online services at the regional and national levels.

Using a mixed-method design, our study will provide valuable insight into the real-life usability and effectiveness of the Inlife intervention. Previous studies have shown that the measures used in intervention studies often do not fit the targets addressed by the intervention [40], therefore, we used a custom interpersonal relationship scale to measure positive interactions, specifically within the dementia care network. Additionally, the semi-structured interviews may provide insight into the possible barriers to and facilitators of implementation. This information will be essential not only to improve the Inlife platform but also to implement the Inlife intervention on a broader scale in clinical practice. Moreover, our study findings will provide recommendations for future research into online interventions for caregivers, as these studies are expected to increase due to the increase in computer literacy and education level of future generations being faced with growing numbers of PwD [11].

In conclusion, the results of this study will add to the knowledge base of the usability and effectiveness of e-health interventions to support caregivers of PwD. We expect that Inlife will effectively increase caregiver social support and feelings of competence, and might aid in the positive interactions within the dementia caregiver network. This study will also provide valuable insights for policy makers, clinicians and researchers into the challenges in the usability and implementation of online social support interventions in the field of dementia care.

Given the promising benefits of technology and online support [9, 11], Inlife might be a valuable social support resource that can be used in routine dementia care. Where the focus is shifting towards improving social health and living well with dementia [41, 42] by maintaining independence and engagement in social activities and daily life for PwD and their caregivers.

### Trial status

The recruitment of participants for this study began in June 2016, and currently, caregivers of PwD are being recruited.

## Additional file

Additional file 1: SPIRIT checklist. (PDF 108 kb)

## Abbreviations

CarerQol: Care Related Quality of Life scale
CONSORT: Consolidated Standards of Reporting Trials
EQ-5D: Euroqol-5D
HADS: Hospital and Anxiety Stress Scale
ICECAP*-*O: Investigating Choice Experiments for the Preferences of Older People Capability Measure for Older People
LS: Loneliness Scale
LSNS*-*6: Lubben Social Network Scale
MRC: Medical Research Council
MSPSS: Multidimensional Scale of Perceived Support
PPQ: Programme participation questionnaire
PSS: Perceived Stress Scale
PwD: People with dementia
RCT: Randomised controlled trial
SPIRIT: Standard Protocol Items: Recommendations for Interventional Trials
SSCQ: Short Sense of Competence Questionnaire
SSL12*-*I: Social Support List 12-Interactions
VAS: visual analogue scale
